# Joint action aesthetics

**DOI:** 10.1371/journal.pone.0180101

**Published:** 2017-07-25

**Authors:** Staci Vicary, Matthias Sperling, Jorina von Zimmermann, Daniel C. Richardson, Guido Orgs

**Affiliations:** 1 Department of Psychology, Goldsmiths, University of London, London, United Kingdom; 2 School of Social Sciences and Psychology, University of Western Sydney, Penrith, New South Wales, Australia; 3 Independent Artist and Choreographer, London, United Kingdom; 4 Department of Experimental Psychology, University College London, London, United Kingdom; University of Western Ontario, CANADA

## Abstract

Synchronized movement is a ubiquitous feature of dance and music performance. Much research into the evolutionary origins of these cultural practices has focused on why humans *perform* rather than *watch* or *listen to* dance and music. In this study, we show that movement synchrony among a group of performers predicts the aesthetic appreciation of live dance performances. We developed a choreography that continuously manipulated group synchronization using a defined movement vocabulary based on arm swinging, walking and running. The choreography was performed live to four audiences, as we continuously tracked the performers’ movements, and the spectators’ affective responses. We computed dynamic synchrony among performers using cross recurrence analysis of data from wrist accelerometers, and implicit measures of arousal from spectators’ heart rates. Additionally, a subset of spectators provided continuous ratings of enjoyment and perceived synchrony using tablet computers. Granger causality analyses demonstrate predictive relationships between synchrony, enjoyment ratings and spectator arousal, if audiences form a collectively consistent positive or negative aesthetic evaluation. Controlling for the influence of overall movement acceleration and visual change, we show that dance communicates group coordination via coupled movement dynamics among a group of performers. Our findings are in line with an evolutionary function of dance–and perhaps all performing arts–in transmitting social signals between groups of people. Human movement is the common denominator of dance, music and theatre. Acknowledging the time-sensitive and immediate nature of the performer-spectator relationship, our study makes a significant step towards an aesthetics of joint actions in the performing arts.

## Introduction

The performing arts form an integral part of human societies. Music and dance are universally practiced and enjoyed across cultures [1,2]. Evolutionary theory provides four possible reasons as to why humans engage in these performative activities. Firstly, dance and music may be by-products of language evolution, as they share structural similarities [3–6], and rely on the same social learning mechanisms, in particular imitation [7,8]. Secondly, choreographed displays of sound and movement aid mate selection in animals, such as the Australian lyrebird [9]. In humans, perceived dance skill is related to both physical strength and male attractiveness [10–12]. Thirdly, performing dance and music might fulfill a social function, as it has been shown that amongst participants, synchronous movement promotes social bonding [13–15]. Finally, synchronous movements may not only promote prosocial behavior in performers, but communicate group cohesion to spectators, signaling ‘coalition quality’ to potential opponents or allies [16,17].

Evolutionary theories on the origins of music and dance often draw on examples from remote and rural communities or rituals such as fire walking [18], but do not easily explain the role of these activities in modern society. Spectators typically don’t expect to mate or form alliances with performers when going to the theatre, yet across Europe, significant numbers of people attend live concerts, dance and opera performances [19]. In this study, we link an evolutionarily relevant feature of group movement to the aesthetic appeal of contemporary western dance performance. We show that dynamic changes in movement synchrony among a group of performers predict continuous measures of spectators’ affective responses to a series of live dance performances.

Synchronizing verbal or nonverbal actions over time is a common feature of group performances [20,21]. Research in social psychology has shown that when people interact with each other, they become more like each other. In conversation, people mimic each other’s facial expression, sway together and look in the same directions [22–24]. This tendency towards entraining to another person’s movements provides a form of ‘social glue’ that enables groups to work together more effectively. People who move together in time, are more likely to remember [25,26] and like each other [27,28], experience feelings of togetherness and similarity [29], cooperate more effectively [30] and are more likely to conform to each other's behavior [31].

In a recent study, we demonstrated that these prosocial effects of behavioral synchronization are related to sustained temporal coupling of similar movements between pairs of people, rather than *unison* group movement, in which the same movements are performed at the same time [32]. Participants in this study performed a set of movement tasks that involved simple movements, such as walking in circles and arm swinging. They either moved synchronously or asynchronously, while we recorded their individual movement dynamics using wrist accelerometers, and quantified their group synchrony using cross-recurrence analysis. Group conformity, mutual liking and affiliation to the group were correlated with sustained coupling of movement dynamics between pairs of people, but did not depend on unison group movement.

Although the pro-social effects of synchronous movement have been well established for those doing the moving, far less is known about the effects on those who might be watching. It has been shown that groups or pairs who successfully move together in time, are perceived as being more socially cohesive relative to groups whose movements are not synchronized [33]. Watching other people act jointly is also rewarding for observers [34]. But what has not been shown is how watching movement synchrony relates to the aesthetic judgment of joint performance. Interestingly, aesthetic perception of orchestral music is related to the leader-follower relationships among the players and their conductor. Musical recordings with misaligned timing of instruments are liked less than correctly aligned musical recordings, and listeners attribute greater skill and closer social bonds to more synchronous players [16], but the role of *visual* synchrony in aesthetic perception of movement has not been studied scientifically. Entrainment to another performer’s movements is a key feature of not only music performance, but also dance [35–37]. In fact, professional dancers are experts in synchronizing their movements with others [38]. Yet, existing experimental research on dance aesthetics has largely focused on the movements of a single performer [5,39–42], the role of motor expertise and movement virtuosity [43–45] or the relationship between movement and sound [46–49].

**Fig 1:**
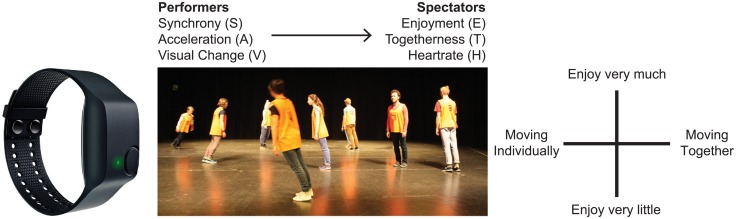
Setup of the experiment. Three time-series were extracted from the performer’s wrist sensors (acceleration/synchrony) and the video recordings (visual change) to predict audience responses recorded from tablet computers (Enjoyment/Togetherness) and wrist sensors (Heart Rate).

To study the role of synchrony in dance aesthetics, we developed a choreography that continuously manipulated movement synchrony among 10 professional dance performers, and based on the movement tasks used in a previous study [32]. During four live performances of this choreography, we equipped both performers and spectators with wrist sensors to quantify performer synchrony, spectator arousal and enjoyment over time (see Fig 1). We combined time-series analysis, used previously in studies of music [50,51], dance [52] and film [53] with cross recurrence analysis of behavioral synchronization [54] to assess nonverbal communication between performers and spectators via observed movement dynamics [55,56].

Specifically, dynamic changes in movement synchrony should predict continuous measures of audience engagement. If synchrony is a key contributor to dance aesthetics with an evolutionary origin, spectator enjoyment and arousal should depend on *how* movements are coordinated in the group, rather than *which* movements are being performed. We therefore related aesthetic appreciation of dance to three dynamic variables, (a) visual motion on stage, as derived from the video recordings of the performances (b) overall performed acceleration and (c) synchrony among performers.

## Methods

### Participants

A total of 101 adults participated as audience members across five live performances (M age = 29 years, SD age = 11.20 years, 33 Males). All participants were paid £10 for participation as an audience member. All participants signed informed consent and the study was approved by the ethical committee at Brunel University London. The breakdown of audience demographic data for performances 2–5 is given in Table 1. The first performance served as a technical pilot, leaving four performances for analyses, hereafter P1-P4. Notably, 32 participants identified as having dance experience, with years of experience ranging from 1 to 45 years across the performances (M = 7.33, SD = 8.35).

**Table 1:** Participant demographics for all four performances.

Performance Session	N Total	N Tablet	Number of Males	Mean Age (Range)	Number with Dance Experience	Mean Dance Experience (Range)
P1	12	11	3	34 (24–60)	5	8.00 (8)
P2	24	15	9	29 (19–65)	7	16.43 (42)
P3	24	10	9	26 (19–39)	9	4.40 (12)
P4	23	15	8	24 (18–42)	11	5.12 (14)

### Performances

A choreography for 10 professional dancers (‘Group Study’) was developed by one of the co-authors, MS (see S1 Video for a short excerpt). Our aim was to create a choreography that would qualify as performative art, yet would focus specifically on the role of movement synchronization in dance aesthetics. To achieve this, we used a movement vocabulary rooted in everyday actions, often associated with contemporary and post-modern dance practices [57,58]. In contrast to other more stylized dance forms such as ballet, this allowed us to maximize variations in movement synchrony, whilst controlling for the influence of familiarity with specific dance styles on the spectators’ aesthetic responses. Experience with stylized dance forms will vary considerably between spectators, whereas pedestrian movements should be equally familiar to all participants [7,45,59,60] Secondly, rather than containing a fixed series of steps, movement sequences were structured by choreographic tasks (see supporting information for the complete choreographic score, S1 Text). For example, performers copied specific movement features (e. g. walking speed or walking direction) from each other over a certain period of time, and mixed moments of near perfect synchrony with moments of asynchronous, independent movement. These choreographic tasks thus allowed us to manipulate movement synchrony independent from movement vocabulary and movement intensity, since the exact movements that performers copied from each other varied within and across performances. In loose analogy to jazz music, the macro-level structure of the choreography and its movement vocabulary were thus matched across all performances. At the same time, smaller scale transitions between movements varied, as they depended on specific performer interactions and decisions. The choreography therefore establishes a performance-specific relation between the performers’ movements and the immediate audiences’ aesthetic and physiological responses to watching these movements. Finally, the choreography was performed without music [49]. Synchrony in this study thus implies entrainment to another performer’s movements, rather than entrainment to an external rhythmical signal [61]. Aesthetic judgements in our study are purely based on the performers’ actions, and not on a combination of the performer’s actions with musical accompaniment.

Following an initial technical pilot on the same day, ‘Group Study’ was performed four times to four different audiences over two days, having an average duration of 32 minutes 44 seconds (range: 28 minutes and 11 seconds to 35 minutes and 4 seconds). All complete performances are available to view at https://www.youtube.com/playlist?list=PLDAYdl_CLkZj2qWBDkxxVug4c5HQmCiB1. All 10 performers signed informed consent and agreed to public dissemination of images and videos of the performances.

### Procedure

The experiment was conducted in a theatre space, with the audience in tiered seating. At the beginning of each performance, all performers and spectators were equipped with Empatica E4 sensors [62] on their left wrist. For the performers, the wrist sensors enabled tracking of acceleration in three-dimensional space. For spectators, the wrist sensors were used to detect heart rate change over time, as an implicit measure of arousal and engagement with the performance. To avoid movement artifacts whilst recording heart rate, audience members were instructed to avoid moving their left arm.

For each performance and in addition to the wrist sensors, 15 audience members were provided with ASUS touchscreen devices, each running the android version of OpenSesame experiment software [63]. A custom finger tracking application was programmed to continuously collect the location of the participant’s index finger on the touch screen. Shown on the touch screen was a two-dimensional grid (white text on black background), with ‘Togetherness’ on the x-axis and ‘Enjoyment’ on the y-axis. (see Fig 1). Togetherness ranged from ‘Moving Individually’ on the far left to ‘Moving Together’ on the far right and gave an estimate of the degree to which the spectator believed the performers were moving as a group. Enjoyment ranged from ‘Enjoy Very Much’ at the top of the screen to ‘Enjoy Very Little’ at the bottom of the screen, as a measure of the spectator’s enjoyment of the choreography over time. Participants were instructed to judge both dimensions independently, and continuously throughout the performance, in the direction most consistent with their current evaluation. For example, if a participant thought the performers were moving together but did not find that part of the performance enjoyable, they should move their finger to the left and downwards. A practice session with the tablets took place before the performance began to allow participants to familiarize themselves with the tablet.

Spectators were informed that they would be seeing a ‘work in progress’ and that their responses would be used to design the final version of the choreography. The purpose of the brief was to establish a realistic performative context and to encourage spectators to use the full range of the enjoyment scale. At the end of the performance, spectators completed a short feedback questionnaire and were debriefed on the purpose of the experiment. Performer and spectator sensors were time-locked and aligned with the audience response data from the tablet. Each performance was filmed from the audience perspective, with a camera positioned behind the audience seating (see S1 Video). These videos were later used to compute an average amount of visual change for each performance. This allowed us to assess aesthetic appreciation as a function of three dynamic variables, (a) visual motion on stage (b) overall performed acceleration and (c) synchrony among performers.

### Data preprocessing

For each performance, we calculated six time-series variables. Three were derived from the performers: their acceleration at each moment in time (averaged over their wrist accelerometers), their synchrony with each other (calculated from a cross recurrence analysis of their acceleration) and the visual change that was produced on screen (derived from video analysis). Three time-series variables were derived from the spectators by averaging their values: their togetherness and enjoyment ratings from the evaluation grid, and their heart rates. The first five minutes of the data were removed from each individual series, to account for the audience adjusting to the rating procedure.

### Acceleration

The acceleration series was extracted from wrist sensors of each of the 10 performers. The magnitude of acceleration was then calculated from the 3-axis acceleration data, by taking the square root of the sum of squared x, y and z values, leaving a single time series vector for each performer. Acceleration was then averaged across all 10 performers within time windows of two seconds. Greater values indicate increases in acceleration.

### Performed synchrony

We applied cross recurrence quantification analysis (CRQA) to the non-windowed acceleration vectors to obtain a continuous measure of synchrony among performers across time. CRQA gives an indication of the degree to which two time-series ‘recur’ or reach a similar point, over time, at different time lags to each other [64]. Cross recurrence was calculated with an embedding dimension of 1, a radius of 10 and a delay of 1 [65]. Whilst other, data driven methods of selecting CRQA parameters exist (e.g. optimise procedure of the CRQA package for R), the sheer number of data points within each data series collected for each performance rendered this procedure implausible. Instead, the same parameter values were chosen for all performances by running the analysis with a series of radius values and deciding upon the value at which the amount of recurrence appeared stable. The resulting percent recurrence rate (%RR) statistic is taken as an approximation of synchrony between two time-series. The recurrence rate was calculated for every possible pair of performers (N = 90 pairs), within a +/- 2 second lag window. The average of all pairs was taken to represent the synchrony of the group; for more details on the calculation of CRQA measures, see [32,54]. This resulted in a single time-series vector representing the percent recurrence rate in non-overlapping time windows spanning 2s, which we term performed synchrony. Higher values of performed synchrony indicate greater synchrony of movement acceleration among the performers.

### Visual change

Visual change was obtained by calculating the pixel-wise change in successive frames of the gray scale version of each performance video, using an established algorithm [45,66,67]. To match the synchrony variable, the time series was recalculated into 2s time windows. Higher values on visual change indicate a greater amount of displacement between successive frames of the video.

#### Enjoyment and perceived synchrony

Enjoyment and togetherness ratings were extracted from each touch screen device, averaged across participants and realigned to represent the same time events as the performer data described above. The raw data for each variable were inspected visually for outliers or erroneous recordings, such as a participant never moving their finger, or moving very infrequently across time. Across performances, the tablet data of six participants were excluded from analyses due to artifacts or technical failure (see Table 1). The enjoyment time series was recorded from the vertical axis of the touch screen, and averaged into two second time windows across participants for each performance. Positive values on enjoyment indicate moments of greater enjoyment, while negative values indicate lesser enjoyment. The perceived synchrony time-series was recorded from the horizontal axis of the touch screen, and processed identically to spectator enjoyment. Positive values indicate greater perceived synchrony among the performers.

### Heart rate

Heart rate data were extracted from the wrist sensors of all audience members. The data were visually inspected for missing data and movement artifacts, which resulted in an impossibly low or high heart rate, with no more than one participant removed for each performance, four in total. The individual heart rate time series were then averaged across participants into 2s time windows and realigned with all other performer and spectator variables.

A data set containing all averaged time-series variables for each performance is publicly available at Goldsmiths public data repository at http://research.gold.ac.uk/20364/

### Statistical analysis

Our primary goal was to determine if synchrony predicts audience engagement with live dance performances. To do this we applied granger causality analysis (GC) to predict spectator heart rate and enjoyment from the three measures of performer motion. GC accounts for the presence of autocorrelations and is able to identify meaningful lagged relationships between two time-series at different timescales [50]. A predictor variable, x, is said to ‘granger cause’ a response variable y, if information about the previous values of x is useful in predicting future values of y, over and above prediction based on information about previous values of y alone [68]. In addition to statistical significance (*p* < .05), we considered GC relationships meaningful and interpretable if two additional criteria were fulfilled. Firstly, GC should be *unidirectional*: Performer variables (e.g. synchrony) should predict audience variables (e. g. enjoyment), but audience variables should not predict performer variables [69]. Secondly, aesthetic responses that result from direct and immediate communication between performers and spectators should be *performance-specific*. Randomly mismatching performer variables from one performance (e.g. P3 synchrony and P4 enjoyment) should abolish significant relationships between performer and spectator variables that exist for variables that are derived from the same performance (P4 synchrony and P4 enjoyment).

Given that aesthetic responses to dynamic art forms such as dance and music are likely to involve a sampling period of at least a couple of seconds [53,70] we assessed granger causal relationships at temporal delays between 2 and 10s. To ensure stationarity [50,51,51], all time-series were differenced by subtracting consecutive sample points from each other (e. g. enjoyment_td_ = enjoymentt2—enjoyment_t1_) prior to applying GC.

Finally, in order to compare the relative contributions of synchrony, overall acceleration and visual motion to the spectators’ enjoyment ratings, we used autoregressive modeling (ARX). As the three performer variables are all derived from the performers’ movement dynamics, we used bivariate rather than multivariate models of enjoyment; the latter assumes independence of the contributing variables. To select the ‘best’ model of enjoyment we compared the baseline (no predictor) model against models that included performer variables, using two information criteria statistics, AICc and BIC. AICc is specifically suited for exploratory models in which not all predictor variables are known [71–73]. BIC is a more conservative measure that penalizes against the inclusion of increasing number of predictors [51]. The best models here should reduce both the AICc and BIC statistics. If communicating group cohesion is aesthetically relevant, synchrony should reduce the information criteria estimates from the baseline model and produce a model that has white noise residuals [50]. Only candidate models that meet these criteria are detailed here.

## Results

**Fig 2:**
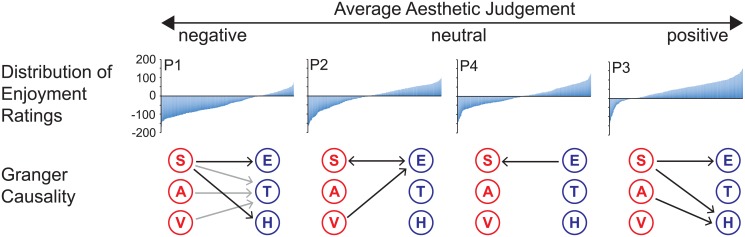
Summary of results. P1 and P3 produced on average positive (P3) or negative (P1) aesthetic judgements, baseline corrected across all performances. For these two performances, enjoyment and heart rate were predicted by synchrony (black arrows). Predictive relationships between the performers movements and perceived togetherness were present only for P1 (grey arrows). (S) Synchrony, (A) Acceleration, (V) Visual Change, (E) Enjoyment, (T) Togetherness, (H) Heart rate.

Results are reported in three parts and separately for all four performances. Firstly, we assess summative aesthetic appreciation and spectator agreement across performances. Then we examine whether participants’ togetherness ratings accurately reflect our objective measures of performer synchrony. Finally, we test the key hypothesis that performer synchrony predicts physiological and subjective measures of spectator engagement, using granger causality analyses. Our key findings are illustrated in Fig 2 and summarized at the end of the results section.

### Overall enjoyment and agreement among spectators

An agreement analysis [52,74] of enjoyment ratings for all spectators showed that approximately 65–70% of all data points occurred within 0.5 standard deviations above the mean of all enjoyment values (P1: 67%, SE = 47.2, P2: 70%, SE = 50.2, P3: 69%, SE = 38, P4: 65%, SE = 41.4). Additionally, the average standard error of the same enjoyment responses for each performance fell within approximately 10% of the possible response range (P1: 11.8%, P2: 12.5%, P3: 9.5%, P4: 10.4%). Together these values indicate good agreement in ratings of enjoyment across audience members for all performances [52,74]. Moreover, there was no significant relationship between mean enjoyment rating and subjective dance expertise, across all performances (*r* = -.19, *p* = 0.22). Accordingly, enjoyment ratings were suitable for averaging across all participants for each performance. A figure showing averaged performer and spectator variables and descriptive statistics for all performances, including Pearson correlations, are provided in the supporting information (S1 Fig, S1 and S2 Tables).

To assess overall differences between performances, we compared the mean enjoyment ratings of all four performances, collapsed across time. A one-way ANOVA showed a main effect of performance on enjoyment, *F*(3, 3302) = 391, *p* < .001, *η*^*2*^ = 0.26. Bonferroni-corrected post-hoc t-tests showed that enjoyment in P3 was significantly greater than in all other three performances (P3 vs. P1: *T*(1688) = 34.77, *p* < .001; P3 vs. P2: *T*(1581) = 17.68, *p* < .001; P3 vs. P4: *T*(1669) = 17.47, *p* < .001). In contrast, P1 showed a significantly lower mean rating than all other performances (P1 vs. P2: *T*(1633) = 15.24, *p* < .001; P1 vs. P4: *T*(1721) = 17.87, *p* < .001). P2 and P4 were not significantly different from each other, *T*(1634) = 1.44, *p* = 0.15. To adjust enjoyment ratings for a general response bias due to using the tablet, we subtracted the global mean of enjoyment across all performances (M = 18.14, SD = 61.7) from the enjoyment time series of each performance. As apparent in Fig 2, audience members of P2 and P4 spent roughly equal amounts of time enjoying or not enjoying the performance, since mean enjoyment did not significantly differ from baseline (*T*(1720) = -1.15, *p* = 0.25, and *T*(1720) = 1.01, *p* = 0.32 respectively). In contrast P1 was overall not enjoyed (*T*(1720) = -24.02, *p* <. 001), whereas P3 was overall enjoyed (*T*(1720) = 26.78, *p* < .001).

To summarize, we observed good agreement among the members of each audience, but found significant differences between audiences. Accordingly, we report detailed time series analyses for all performances separately, focusing on acceleration, visual change and synchrony in relation to spectator enjoyment and heart rate.

### Granger causality analyses

Since our hypothesis focusses on live communication between performers and spectators, we examine lags between -10 and +10 seconds. Positive lags indicate performer variables predicting spectator variables, negative values indicate spectator variables predicting performer variables. We consider a relationship between variables predictive only if it is *unidirectional*. Secondly, we controlled for the presence of spurious relationships in our data by computing GC between performer and spectator time series of the same performance, and comparing these to GC relationships derived from randomly mismatched performances. For mismatching, P2 was paired with P1, P3 with P2, P4 with P3 and P1 with P4. We only interpret GC relationships that are *performance-specific*.

### Performed synchrony and perceived ‘togetherness’

We measured *performed* synchrony as cross-recurrence of acceleration profiles between performers. This measure may or may not reflect the spectator’s notion of what “moving together” means. We therefore investigated the link between *performed* synchrony and *perceived* synchrony, relating cross-recurrence of acceleration profiles to the spectators’ ratings of togetherness for all performances.

A relationship between performed and perceived synchrony existed only for P1 with a lag of 2s, *F*(1, 856) = 4.49, *p* = 0.03, 6s, *F*(3, 852) = 2.76, *p* = 0.04 and 10s, *F*(5, 848) = 3.2, *p* = 0.007. These relationships were both unidirectional and performance specific (all *p* > .05). Interestingly, for P1 overall acceleration and visual change also predicted ratings of togetherness at a lag of 2–10s, acceleration: *F*(1, 856) = 4.03, *p* = 0.045, *F*(3, 852) = 5.39, *p* = 0.001, *F*(4, 850) = 4.29 *p* = 0.002, *F*(5, 848) = 3.67, *p* = 0.003), and at a lag 6 to 10 s; visual change: (*F*(3, 852) = 3.41, *p* = 0.02, *F*(4, 850) = 2.63, *p* = 0.03, *F*(5, 848) = 2.33, *p* = 0.04). These relationships were both unidirectional and performance-specific (all *p* > .05). There were no significant GC relationships between any of the performer variables and perceived synchrony for P2, P3 or P4 (Fig 2).

These findings indicate a dissociation between performed synchrony on stage and the spectators’ interpretation of what ‘moving together’ means. Ratings of togetherness were either completely unrelated to measures of behavioral synchronization, or they were equally associated with measures of acceleration and visual change, rather than performer synchronization only.

### Performed synchrony, enjoyment and heart rate

If audience engagement is dynamically linked to movement synchronization among performers, we should observe significant, unidirectional and performance-specific GC of synchrony predicting subjective and physiological measures of audience engagement. Results are reported separately for all performances.

#### P1

**Fig 3:**
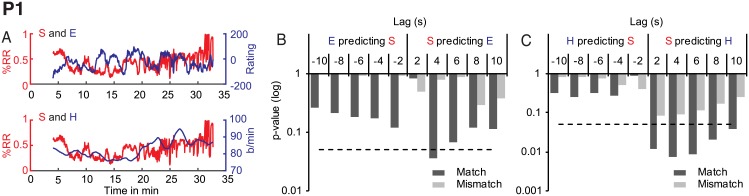
P1 Granger causality results for synchrony (S), enjoyment (E) and heart rate (H). A. Overlayed time-series for synchrony and enjoyment (top panel) and synchrony and heart rate (bottom panel). B. GC for synchrony and enjoyment. C: GC for synchrony and heart rate. The dashed line indicates a significance level of *p* < .05.

For P1, synchrony predicted enjoyment at a lag of 4 s, *F*(2, 854) = 3.32, *p* = 0.04 (Fig 3A and 3B). This relationship was unidirectional, with no significant reciprocal granger causality of enjoyment on synchrony and it disappeared when we randomly used P2 synchrony to predict P1 enjoyment (*p* at all lags > .05). Similarly, there were no significant GC relationships between either acceleration or visual change and enjoyment.

Synchrony was also granger causal of heart rate with a delay of 2-10s (F(1, 856) = 6.4, p = 0.01, F(2, 854) = 4.90 *p* = 0.007, *F*(3, 852) = 3.92 *p* = 0.008, *F*(4, 850) = 2.90, *p* = 0.02 and *F*(5, 848) = 2.35, *p* = 0.04), see Fig 3A and 3C. This relationship was unidirectional, with no significant GC of heart rate on synchrony and performance-specific; mismatching P1 heart rate with P2 synchrony produced no significant effects (all *p* > .05). We observed no other significant influences of any of the performer variables on any of the spectator variables.

#### P2

**Fig 4:**
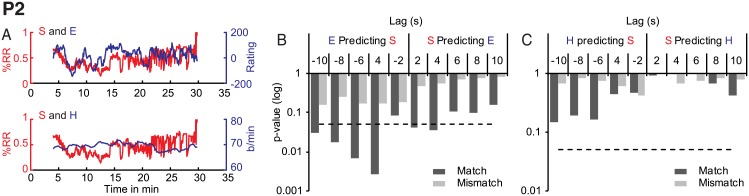
P2 Granger causality results for synchrony (S), enjoyment (E) and heart rate (H). A. Overlayed time-series for synchrony and enjoyment (top panel) and synchrony and heart rate (bottom panel). B: GC for synchrony and enjoyment. C. GC for synchrony and heart rate. The dashed line indicates a significance level of *p* < .05.

We observed significant granger causality between synchrony and enjoyment at a lag of 2s, *F*(1, 769) = 4.21, *p* = 0.04 and 4s, *F*(2, 767) = 3.34, *p* = 0.04, see Fig 4A and 4B. However, these effects were not unidirectional, since enjoyment was equally predictive of synchrony at all lags except -2 s, (*F*(2, 767) = 5.96, *p* = 0.003, *F*(3, 765) = 4.12, *p* = 0.007, *F*(4, 763) = 3.02, *p* = 0.02, and *F*(5,761) = 2.49, *p* = 0.03). Instead, enjoyment was significantly predicted by visual change, with a delay of 6-10s, *F*(3, 765 = 6.44, *p* = 0.0003, *F*(4,763) = 5.21, *p* = 0.0004, *F*(5, 761) = 4.18, *p* = 0.0009. This relationship was both unidirectional and performance-specific (all *p* > .05). We observed no other significant influences of any of the performer variables on any of the spectator variables.

#### P3

**Fig 5:**
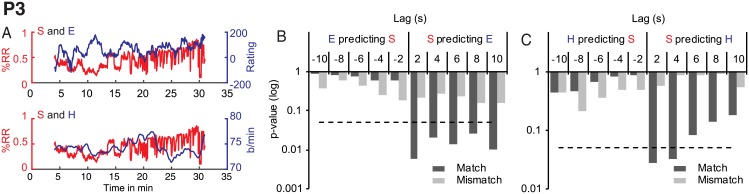
P3 Granger causality results for synchrony (S), enjoyment (E) and heart rate (H). A. Overlayed time series for synchrony and enjoyment (top panel) and synchrony and heart rate (bottom panel). B: GC for synchrony and enjoyment. C. GC for synchrony and heart rate. The dashed line indicates a significance level of *p* < .05.

Synchrony was granger causal of enjoyment at all tested lags, *F*(1, 804) = 7.65, *p* = 0.006, *F*(2, 802) = 3.87, *p* = 0.02, *F*(3, 800) = 3.55, *p* = 0.01, *F*(4, 798) = 2.76, *p* = 0.03, and *F*(5, 796) = 3.04, *p* = 0.01, indicating prediction of enjoyment within a delay of 2–10 seconds, see Fig 5A and 5B. This relationship was unidirectional, with no significant relationships in the reverse direction, and performance-specific (all *p* > .05). P4 synchrony did not predict P3 enjoyment.

Moreover, synchrony predicted heart rate at a lag of 2–4 s, *F*(1, 804) = 4.8508, *p* = 0.03, *F*(2, 802) = 3.43, *p* = 0.03). This relationship was both unidirectional and performance specific (all *p* > .05), see Fig 5A and 5C.

For P3 only, we observed an additional relationship between overall acceleration and heart rate with a delay of 2–8 s (*F*(1, 804) = 4.43, *p* = 0.04, *F*(2, 802) = 4.6, *p* = 0.01, *F*(3, 800) = 3.25, *p* = 0.02, *F*(4, 798) = 2.71, *p* = 0.029. This relationship was unidirectional and performance specific with no influence of P4 acceleration on heart rate or vice versa (all *p* > 0.05). We observed no other significant influences of any of the performer variables on any of the spectator variables.

#### P4

**Fig 6:**
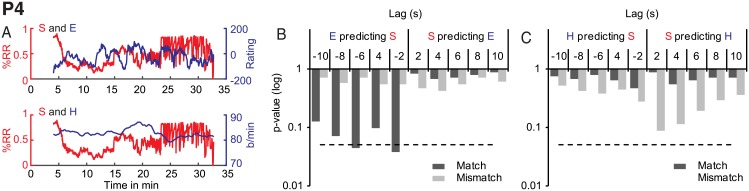
P4 Granger causality results for synchrony (S), enjoyment (E) and heart rate (H). A. Overlayed time series for synchrony and enjoyment (top panel) and synchrony and heart rate (bottom panel). B: GC for synchrony and enjoyment. C: GC for synchrony and heart rate. The dashed line indicates a significance level of *p* < .05.

For P4 we did not observe any significant GC relationships of synchrony, acceleration or visual change on either enjoyment or heart rate (all *p* > 0.05). However, similar to P2, enjoyment was predictive of synchrony at 2 s and 6 s, *F*(1, 857) = 4.3035, *p* = 0.04, *F*(1, 853) = 2.6936, *p* = 0.045, see Fig 6A and 6B. This seemingly paradoxical relationship was also performance-specific. P1 enjoyment did not predict P4 synchrony. We observed no other significant influences of any of the performer variables on any of the spectator variables.

To summarize, granger causality analyses provided consistent evidence for a direct link between synchrony and audience engagement for those performances that were either overall enjoyed (P3) or not enjoyed (P1). In contrast, for P2 and P4 granger causal relationships were spurious or absent between these two variables. Interestingly, spectators of P2 and P4 spent on average equal amounts enjoying and not enjoying what they saw, suggesting that spectators did not form a consistent aesthetic response in either positive or negative directions over the course of these two performances.

### ARX modelling

In order to compare the relative contributions of synchrony, overall acceleration and visual motion to the spectators’ enjoyment ratings, we applied autoregressive modeling (ARX). The strongest case for a role of behavioral synchronization in aesthetic perception of dance would be made if synchrony improved ARX models of enjoyment (a) consistently across all performances and (b) to a greater degree than either acceleration or visual change (c) in a performance specific manner, with no influence of synchrony from a randomly mismatched performance.

Table 2 shows that for three out of four performances, synchrony improved the best autoregressive model of enjoyment, as measured by AICc. Consistent with the GC results, P1 contributed at lag 2 and P3 contributed at lag 1. Importantly, acceleration did *not* contribute to the model for any of the four performances; in fact the inclusion of acceleration at times resulted in worsened AICc statistics than the autoregressive model alone. In line with our GC analyses, visual change contributed to the model of enjoyment only for P2 and was the primary predictor of enjoyment within the model. All ARX models of enjoyment were improved by movement synchronization including up to 10 seconds of prior information sampling. The improvement in prediction of audience enjoyment with the inclusion of performed synchrony explained on average 10% of the variance of the model of enjoyment. Consistent with the granger test results, synchrony from a mismatched performance did not improve the model of enjoyment for any of the performances.

**Table 2:** ARX model results.

	Modeled Variable	Model Autoregressive (ar) and Predictor inputs (lags)	AICc	Change in AICc	BIC	Estimate of data explained
P1	dEnjoy	ar (1, 16)	6225.09		6239.29	9.15%
	dEnjoy	dSynchrony (2), ar(1, 16)	6222.40	-2.69	6241.33	9.65%
	dEnjoy	dVisChange (2), ar(1, 16)	6223.37	-1.72	6242.30	9.54%
P2	dEnjoy	ar(1,2,17,18)	5589.92		5613.02	14.90%
	dEnjoy	dSynchrony (0), ar (1,2,17,18)	5585.17	-4.78	5612.87	15.65%
	dEnjoy	dVisChange (3), ar(1, 2,17,18)	5579.30	-10.62	5607.00	16.30%
P3	dEnjoy	ar (1,2,4,24)	5840.59		5863.91	8.41%
	dEnjoy	dSynchrony (1), ar (1,2,4,24)	5835.81	-4.78	5863.78	9.19%
	dEnjoy	dVisChange (4), ar (1,2,4,24)	5840.07	-0.52	5868.05	8.86%
P4	dEnjoy	ar (1,3,14,17)	6561.16		6589.53	7.32%
	dEnjoy	dPFSync (3), ar (1,3,14,17)	6562.79	+1.63	6595.88	7.71%
	dEnjoy	dVisChange(2),ar (1,3,14,17)	6560.61	-0.55	6588.98	7.38%

### Summary

Our findings show that spectators’ positive and negative overall aesthetic evaluations were associated with performance-specific and time-sensitive relationships between synchrony and explicit (enjoyment ratings) and implicit (heart rate) affective responses. If spectators did not form a strong and stable aesthetic evaluation, relationships between synchrony and enjoyment were spurious, or even reversed, without any influence of synchrony on spectator arousal. Performer synchrony did not easily map onto spectator’s judgements of perceived togetherness, yet proved to be a more consistent predictor of audience engagement then movement acceleration or visual motion, pointing to an important role of behavioral coordination in dance aesthetics (see Fig 2).

## Discussion

Synchronous behaviour pervades everyday life [23,24] and the performing arts [14,15,20]. Here, we tested whether the social signals conveyed by synchronous movement [16,27,75–77] translate to the continuous aesthetic appreciation of live dance performances.

### Dynamic aesthetics of movement synchrony

Behavioral coordination among performers predicts both physiological measures of spectator arousal and subjective reports of enjoyment, but only if spectators form an overall positive or negative aesthetic evaluation of the choreography. Specifically, for spectators of P1 and P3, changes in movement coupling between performers predict changes in enjoyment and heart rate. Overall visual motion and acceleration measures do not reliably predict enjoyment or heart rate. Thus, spectators’ enjoyment is sensitive to *how* performers coordinate their movements, rather than *how much* the performers move. However, synchrony does not predict audience engagement with P2 and P4, and is associated with a summative aesthetic judgement that does not differ from baseline. Importantly, overall differences in summative aesthetic judgements between performances are not easily explained by corresponding overall differences in performer variables, nor did they depend on the spectator’s reported dance expertise. Descriptive measures of all three performer variables are similar across all four performances, yet produce significantly different aesthetic outcomes. We propose that the formation of an overall positive or negative aesthetic evaluation is thus associated with specific time-sensitive relationships between the performer’s movements and audience engagement. In contrast, summative aesthetic judgements that do not differ from baseline imply a lack of information exchange, or a seemingly paradoxical flow of information from spectators to performers.

### Positive and negative affective responses to synchrony

Since GC analyses are performed on differenced time-series, our study does not allow us to make strong claims as to whether synchrony increases or decreases enjoyment. The direction of this influence can vary over the time course of the performance. Correlations between synchrony and enjoyment ratings however suggest that on average, this relationship is overall positive in P3 and negative in P1, in line with the mean aesthetic judgements which were positive for P3 and negative for P1 (S2 Table). Yet, correlations between time-series are not necessarily appropriate to capture the complexity of these directional relationships. This is because synchrony and enjoyment are time-dependant, and so is the direction of their relationship [69]. An overall positive correlation does not mean that the relationship between synchrony and enjoyment was consistently positive throughout the performance. Time-series analyses are better suited to capturing these changes between two variables and allow to predict (through granger causality) one variable from the other.

Enjoying synchrony or asynchrony might also depend on the specific movements that are being performed. For example, spectators prefer movements with extreme velocity profiles such as jumps or quick turns [42,43], movements that they have learnt to perform themselves [45] or movements with fluent movement dynamics [5,39]. Finally, the direction of the relationship between synchrony and aesthetic appreciation should depend on the choreographic structure itself. Extended periods of asynchrony or synchrony, might simply become boring. In line with these ideas, our findings show that enjoyment can increase or decrease with synchrony. Crucially, in both cases it is *predictive* of the aesthetic experience and the spectator’s physiological reaction to the observed movements.

Our findings are consistent with a prominent role of performer-spectator communication in performing arts aesthetics [1,55,56]: Predictive relationships between the performers’ movements and the audience’s responses are associated with the formation of an overall positive or negative mean aesthetic judgement. Conversely, the lack of information flow between performers and spectators is associated with enjoyment ratings that are decoupled from the performers movements, and are equally often negative as they are positive.

Our findings have implications for theories on the evolutionary origins of dance and music. The social bonding hypothesis focuses exclusively on affiliation among performers [14,15] and therefore does not explain why changes in synchrony predict spectator affect. Similarly, dance as an aid for mate selection focuses on individual performers only and cannot explain the importance of joint movement dynamics for aesthetic appreciation. Our findings are however consistent with the ‘coalition signalling’ hypothesis [16]. Participating in group performances does not only produce pro-social effects among performers, but signals social cohesion to spectators [33,34]. These social signals are transmitted via the dynamics of group movement and produce both positive and negative aesthetic outcomes; in both cases, affective responses are temporally coupled to the performers’ movements.

The coalition signalling hypothesis might help to specify contexts in which synchrony increases or decreases enjoyment. If synchrony is a signal of coalition quality, affective responses elicited by watching synchronized movements should depend on how spectators relate to the performing group [16]. Specifically, ‘allies’ should enjoy synchrony, whereas ‘opponents’ should enjoy lack of synchrony. Future studies might test these possibilities by linking aesthetic judgements of synchrony to the extent to which spectators identify with the performing group.

### Performing and perceiving synchrony

In addition to enjoyment ratings, we collected togetherness ratings as a subjective measure of synchrony among performers. Surprisingly, performed and perceived synchrony were not consistently related in our study. Importantly, our measure of performed synchrony is purely acceleration-based and does not capture *spatial* aspects of synchronous behavior. For example, movement acceleration does not inform about the location of the performers’ movements. Secondly, visual synchrony is relatively fuzzy, at least in comparison to auditory synchrony in music. Synchronous movements in dance are less strictly aligned in time. For different bodies to move visually synchronous, their movement acceleration will need to be slightly different. A dancer with longer arms will need to move faster than a dancer with shorter arms in order to arrive in the same position simultaneously. Thirdly, we computed synchrony in 2s time-windows. This implies that moments of unison movement, as well as more loosely coupled joint and complementary movements, which do not necessarily imply identical trajectories, contribute to performed synchrony.

It is possible that explicit judgements of synchrony are more closely linked to these spatial indicators of behavioural coordination, or reflect unison movement only. However, our findings suggest an alternative explanation. Specifically, moments of stillness or collective stopping are associated with high values of cross recurrent acceleration. Yet ‘stopping together’ may not necessarily comply with an intuitive definition of ‘moving together’. Indeed, acceleration correlates positively with togetherness (more movement, more togetherness), but negatively with performed synchrony (less movement, more synchrony) across performances (S2 Table), suggesting that stopping together is indeed an important contributor to performed synchrony.

Despite a clear link between acceleration-based synchrony and togetherness ratings, performed synchrony significantly influenced audience enjoyment, suggesting that spectators were aesthetically sensitive to the coupling of movement acceleration among performers. The lack of a predictive relationship between performed and perceived synchrony suggests that the influence of behavioral coordination on enjoyment occurred *spontaneously*, rather than as a result of spectators explicitly searching for instances of high and low levels of togetherness in the choreography. Although judging togetherness may have primed participants to focus on group movement, such priming can therefore not explain the predictive relationships between performed synchrony and enjoyment in our study.

As is the case when two people perform joint actions such as jumping [78] or manipulating the same object together [79], the spectators’ aesthetic judgements were sensitive to the dynamic characteristics of collective movement. Our findings thus emphasise the importance of dynamic movement parameters in action perception [80–82], emotion recognition [83,84] and extend their relevance to the aesthetic appreciation of the performing arts [42].

### Aesthetic appreciation of live performing arts

Movement is the common denominator of all performing arts, including dance, theatre and music. To highlight the role of synchrony and joint action in aesthetic perception of movement, we chose a movement vocabulary rooted in everyday activities, and did not specify a fixed sequence of specific movement transitions. These principles are often associated with contemporary performing dance practice [57,58] and allowed us to (a) manipulate movement synchrony independent of movement vocabulary and (b) reduce the influence of familiarity with the observed movement on aesthetic perception. Our findings therefore apply to many ritualistic forms of dance and performance situations, that use a similarly ‘casual’ movement material, including social dancing or participatory performances, such as flash-mobs or even live concerts. In more stylised forms of dance, such as western ballet or Indian classical dance, movement aesthetics should additionally depend on prototypicality [40] and familiarity with the movement vocabulary [45], as well as the grammatical structures that govern the transitions between specific movements [4,5]. Finally, dance aesthetics will be strongly influenced by music preferences [49]. Yet, synchrony is ubiquitous across stylised and non-stylised dance practices and in music performance, suggesting that synchrony may be a universal feature of the performing arts with an evolutionary origin: Synchrony allows to efficiently signal coalition quality among a group of performers to a group of spectators [16,17]

A defining feature of the performing arts in particular is that live performances are never identical. While paintings or sculptures rarely change as a result of being watched, live performances often directly depend on immediate interactions with the audience [55,85]. It is therefore likely that some of the performance-specific findings we observe are linked to the contextual specifics of the live performance environment in which our experiment took place. We observed clear differences in summative aesthetic judgement between performances, despite similar performance characteristics and randomly selected audiences. Watching a live performance produces greater audience engagement than watching a recorded version of the same performance [86] and involves social interactions between spectators [85,56]. Research in music aesthetics shows that listeners’ aesthetic appreciation of a tune depends on the availability of social feedback. Participants tend to agree with arousal and valence ratings of others [87]. Available social feedback from other audience members (e. g. chuckling) may have contributed to differences between our performances. Agreement analyses for each performance showed consistent aesthetic responses *within* an audience, despite significant differences *between* audiences, suggesting that audience members may have influenced each other’s aesthetic judgements to some extent. Further, we observed no significant relationship between the spectators’ dance experience and their enjoyment ratings, suggesting that spectator expertise did not mediate aesthetic differences between performances [88]. Despite these challenges for an experimental approach to live performing arts aesthetics, we observed consistent relationships between performed synchrony and audience engagement, if the audience formed a strong and stable aesthetic evaluation of the performance that they were watching.

## Conclusions

Music, dance and theatre differ from other art forms not only in that they involve direct interactions between performers and spectators, but also in that they extend over time. Aesthetic judgements of dance and music will reflect past, present and expected future events of the performance [89]. Existing research in aesthetics has largely focused on discrete aesthetic judgements that cannot capture the dynamic nature of the performing arts. Here, we show that the summative aesthetic appreciation of a performance is related to specific time-sensitive relationships between the performers’ movements and the spectator’s response to these movements. Our study demonstrates that some of the aesthetic appeal of the performing arts lies in communicating cooperation within a group of performers to a group of spectators. Across societies, dance and music fulfil many functions, ranging from courtship and celebration to worshipping gods and ancestors [1]. Our study provides direct experimental evidence for the transmission of social information in the performing arts via collective movement dynamics [56,90].

## Supporting information

S1 Fig Averaged performer (red) and spectator (blue) variables for all four performances. (S) Synchrony, (A) Acceleration, (V) Visual Change, (E) Enjoyment, (T) Perceived togetherness, (H) Heart rate.
(EPS)

S1 Table Descriptive statistics for all four performances. (DOCX)

S2 Table Pearson correlations for all performer and spectator variables. (A) Acceleration, (V) Visual Change, (S) Performed synchrony, (T) Perceived togetherness, (E) Enjoyment, (H) Heart rate, * = p <.01, ** = p <.001, *** = p <.0001.
(DOCX)

S1 Video Excerpt of P3. The video excerpt shows gradual synchronization of the group, tipping over into walking/running. Videos of all four performances are available to view at https://www.youtube.com/playlist?list=PLDAYdl_CLkZj2qWBDkxxVug4c5HQmCiB1.
(M4V)

S1 Text Choreographic score. The choreographic score describes the movement tasks for performers in detail. The score should be used according to the following guidelines.
Don’t add anything to the score that isn’t there.Don’t take away anything from the score that is actually specified.The score specifies parameters of movement, not specific movements.The score specifies contexts in which decisions take place, not the decisions themselves.The score does not specify set durations for the tasks. Rather, task durations are the results of the performers balancing the functional demands of each task with their artistic sensitivity to composition in time.Synchrony in this score is defined as movements with very similar 3D-acceleration profiles, that are coupled in time.This score was developed to produce dynamic variations in synchrony over time, keeping all other aspects of the choreography relatively constant.Performances resulting from this score should be suitable for experimental purposes, but should qualify as performative art.Performances resulting from this score should never be identical, yet should always be clearly identifiable as resulting from the same score.If you are unsure how to implement the score or have any related questions, please contact Matthias Sperling at info@matthias-sperling.com.
(PDF)
