# Supplementary material for: Joint action aesthetics
Source: PLoS One. 2017 Jul 25;12(7):e0180101. doi: 10.1371/journal.pone.0180101 (PMC5526561; doi:10.1371/journal.pone.0180101)
Supplement: S1 Table — (DOCX) [file pone.0180101.s002.docx]

|  | **Acceleration** | | | **Visual Change** | | | **Performed Synchrony** | | |
| --- | --- | --- | --- | --- | --- | --- | --- | --- | --- |
|  | M (SD) | Min | Max | M (SD) | Min | Max | M (SD) | Min | Max |
| **P1** | 1.91 (11.66) | -9.39 | 35.55 | 1.04 (0.57) | 0.21 | 2.56 | 43.37 (16.96) | 11.32 | 99.48 |
| **P2** | 2.08 (10.48) | -8.77 | 35.39 | 1.04 (0.57) | 0.21 | 2.79 | 46.59 (15.56) | 14.01 | 100 |
| **P3** | 2.36 (11.43) | -9.68 | 36.85 | 1.54 (0.81) | 0.21 | 3.53 | 44.51 (16.75) | 9.91 | 85.91 |
| **P4** | 1.67 (11.05) | -9.52 | 36.47 | 1.16 (0.55) | 0.21 | 2.71 | 47.81 (21.06) | 1.55 | 89.42 |
|  | **Enjoyment** | | | **Heart Rate** | | | **Enjoyment** | | |
|  | M (SD) | Min | Max | M (SD) | Min | Max | M (SD) | Min | Max |
| **P1** | 164.02 (227) | -306.06 | 492.15 | -24.91 (52.60) | -150.01 | 96.1 | 82.70 (4.83) | 75.81 | 94.75 |
| **P2** | 125.61 (160.9) | -228.96 | 436.53 | 16.06 (56.12) | -144.85 | 118.04 | 69.38 (1.18) | 66.83 | 71.77 |
| **P3** | 113.63 (167.2) | -167.04 | 454.41 | 64.09 (51.95) | -117.16 | 180.95 | 74.20 (1.29) | 71.37 | 77.41 |
| **P4** | 82.57 (187.4) | -289.77 | 452.2 | 19.89 (51.45) | -123.01 | 144.85 | 82.85 (1.70) | 79.26 | 87.16 |
