# Supplementary material for: Joint action aesthetics
Source: PLoS One. 2017 Jul 25;12(7):e0180101. doi: 10.1371/journal.pone.0180101 (PMC5526561; doi:10.1371/journal.pone.0180101)
Supplement: S2 Table — (A) Acceleration, (V) Visual Change, (S) Performed synchrony, (T) Perceived togetherness, (E) Enjoyment, (H) Heart rate, * = p <.01, ** = p <.001, *** = p <.0001. (DOCX) [file pone.0180101.s003.docx]

| **P1** | **A** | **V** | **S** | **T** | **E** | **H** |
| --- | --- | --- | --- | --- | --- | --- |
| **A** | - | .33 *** | -.76 *** | .50 *** | .04 | -.43 *** |
| **V** |  | - | -.55 *** | .51 *** | .26 *** | -.29 *** |
| **S** |  |  | - | -.61 *** | -.21 *** | .43 *** |
| **T** |  |  |  | - | .39 *** | -.78 *** |
| **E** |  |  |  |  | - | -.11 *** |
| **H** |  |  |  |  |  | - |
| **P2** | **A** | **V** | **S** | **T** | **E** | **H** |
| **A** | - | .07 | -.78*** | .36*** | -.25*** | .09*** |
| **V** |  | - | -.28*** | .23*** | .10*** | .20*** |
| **S** |  |  | - | -.31*** | .30*** | -.001 |
| **T** |  |  |  | - | .09*** | .12*** |
| **E** |  |  |  |  | - | .007 |
| **H** |  |  |  |  |  | - |
| **P3** | **A** | **V** | **S** | **T** | **E** | **H** |
| **A** | - | .39 *** | -.74*** | .49*** | -.05 | -.06 |
| **V** |  | - | -.50*** | .46*** | -.28*** | .13*** |
| **S** |  |  | - | -.45*** | .20*** | .09** |
| **T** |  |  |  | - | .02 | .02 |
| **E** |  |  |  |  | - | -.24 |
| **H** |  |  |  |  |  | - |
| **P4** | **A** | **V** | **S** | **T** | **E** | **H** |
| **A** | - | .37*** | -.79*** | .45*** | .12*** | .10*** |
| **V** |  | - | -.57*** | .41*** | -.15*** | .62*** |
| **S** |  |  | - | -.42*** | .009 | -.30*** |
| **T** |  |  |  | - | .40*** | .33*** |
| **E** |  |  |  |  | - | -.27*** |
| **H** |  |  |  |  |  | - |
