# Supplementary material for: Joint action aesthetics
Source: PLoS One. 2017 Jul 25;12(7):e0180101. doi: 10.1371/journal.pone.0180101 (PMC5526561; doi:10.1371/journal.pone.0180101)
Supplement: S1 Text — The choreographic score describes the movement tasks for performers in detail. The score should be used according to the following guidelines. Don’t add anything to the score that isn’t there.Don’t take away anything from the score that is actually specified.The score specifies parameters of movement, not specific movements.The score specifies contexts in which decisions take place, not the decisions themselves.The score does not specify set durations for the tasks. Rather, task durations are the results of the performers balancing the functional demands of each task with their artistic sensitivity to composition in time.Synchrony in this score is defined as movements with very similar 3D-acceleration profiles, that are coupled in time.This score was developed to produce dynamic variations in synchrony over time, keeping all other aspects of the choreography relatively constant.Performances resulting from this score should be suitable for experimental purposes, but should qualify as performative art.Performances resulting from this score should never be identical, yet should always be clearly identifiable as resulting from the same score.If you are unsure how to implement the score or have any related questions, please contact Matthias Sperling at info@matthias-sperling.com. (PDF) [file pone.0180101.s005.pdf]

Matthias Sperling 'Group Study' (2015): a choreographic score for ten performers

Choose where you will enter from, arranging yourselves in an arc around the edge of the performance space, as indicated in the drawing below. Your individual places in the arc are not predetermined, but the general shape and location of the arc is known.

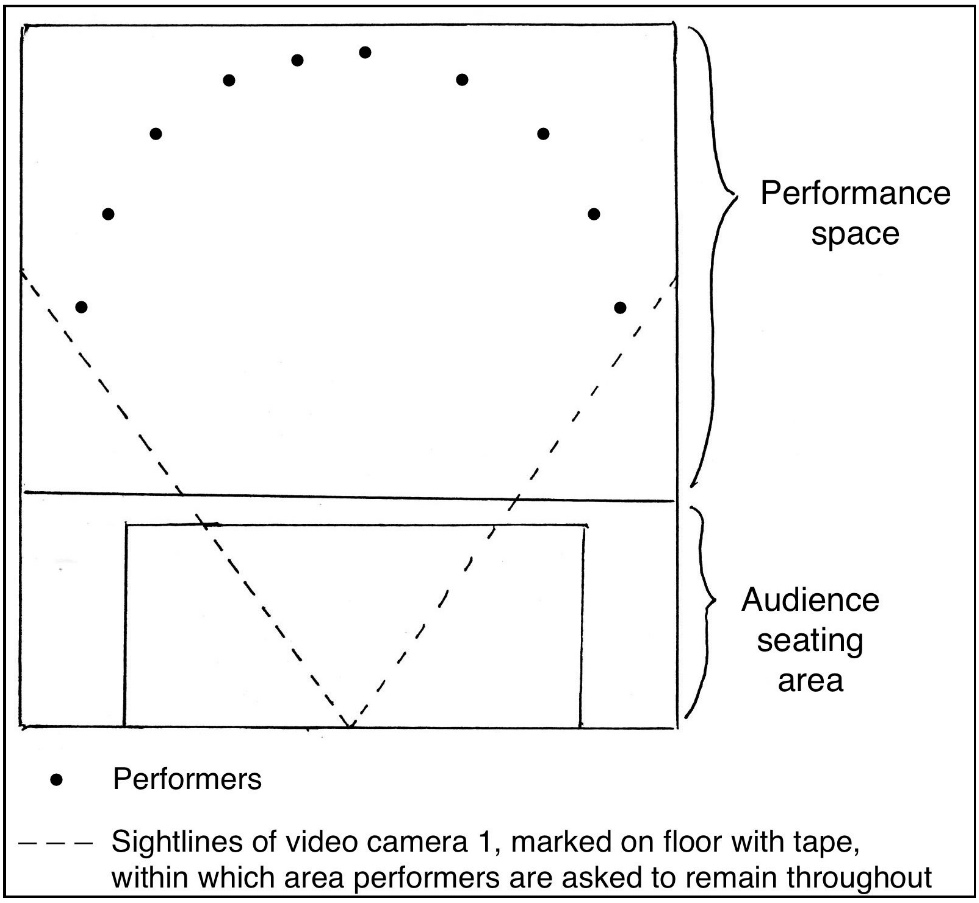

Pouring in:

Using three people as a guide (whoever is at either edge of the arc, and whoever is at the centre), invite the possibility of a shared breath rhythm: on the third outbreath, pour in. Your swift falling walk begins and ends at precisely the same moment as everyone else. You pour into a stillness that is on the way to somewhere, an in-between place that 'what lights up'[1] can contribute to.

Nothing is happening:

You sustain the stillness of the space, while allowing your seeing to remain active. You offer 'nothing is happening' to the audience.

Pickpocketing:

Subtly and sparsely, pickpocketing begins to be introduced. You select and take on another's precise body posture and direction in space, making and unmaking quiet visual patterns. You allow time for the audience to notice this. You move efficiently and without disturbing the air molecules that surround you.

While pickpocketing continues, you begin to count down from ten to one, in a voice that is neither loud nor soft, naming a selection from what is alive in your perception. As you count, an accent could increasingly reveal itself to be the particular way of speaking of the land inhabited by what you are noticing. The intervals between numbers is irregular, with the frequency of your counting increasing in the mid-range, before stretching out again somewhat as you approach your last few numbers. No more than two times, someone in the group uses the 'wildcard' of choosing to walk a few steps to another location, refreshing your landscape.

Looping:

Your count 'one' is your imperceptible launch into looping. You allow what lights up in the feedback from your body to become the origin of a tiny repetitive movement including a weight shift. A little at a time, it extends to include the whole of your articulate, alert body in the process of noticing this movement becoming itself. Its particular shape, rhythm, energy, and spatial behaviour become increasingly apparent to you. Once fully formed, it develops a state of change that is as gradual and continuous as possible, while always insisting on following what interests and enlivens you. During your looping, your seeing has the opportunity to expand and contract, and your activity has the opportunity to have high and low intensity. At least once, you notice an opportunity to direct your activity to join the shape of another's looping, sharing connection for a time, before continuing individually. Your looping is supported by a continuous 'mmmmm' sound that is responsive to your movement. When you have the sense that everyone has had the opportunity to join another, you very gradually allow your loop to approach twisting.

Twisting:

The group meets in the twisting[2], without allowing synchronous timing to arise, and while gathering close together in space with a shared direction of facing towards one of the downstage corners. The location of the gathering is anywhere but the centre, within the downstage half of the performance space. Once collected, small but clear variations in the twisting are introduced, spreading like viruses through the group. Each variation has a vocal sound that matches it closely. As a group, you repeatedly test how close you can get to synchrony without actually getting there; each time you approach complete synchrony, you collectively find ways to disrupt its arrival. One of the variations includes a smooth change of the group's direction of facing. You trust your count of about five variations that have successfully spread to a large part of the group, and choose to return to the twisting action that you began with. With the arrival of this action, you allow complete synchrony to be established.

Countdown/scatter:

While continuing unison twisting, you count down with your individual set numbers[3] from 10. After 5 and before 1, the scattering is triggered by anyone who chooses to. Don't be the last one in the twisting spot.

Circling:

Walking at medium speed, allow two circles rotating in opposite directions to form. Let them be medium-sized, spatially separate circles, made up of roughly equal numbers of people. Continuing to share the medium walking speed, negotiate moving the location of

the circles until one is inside the other. When the circles are concentric, allow variations in the speed to begin to arise (in group circles, let bigger circles generally be faster, and smaller circles slower). Allow the two circles to negotiate separating from each other spatially again. An example of this progression of relationships between the circles is drawn below.

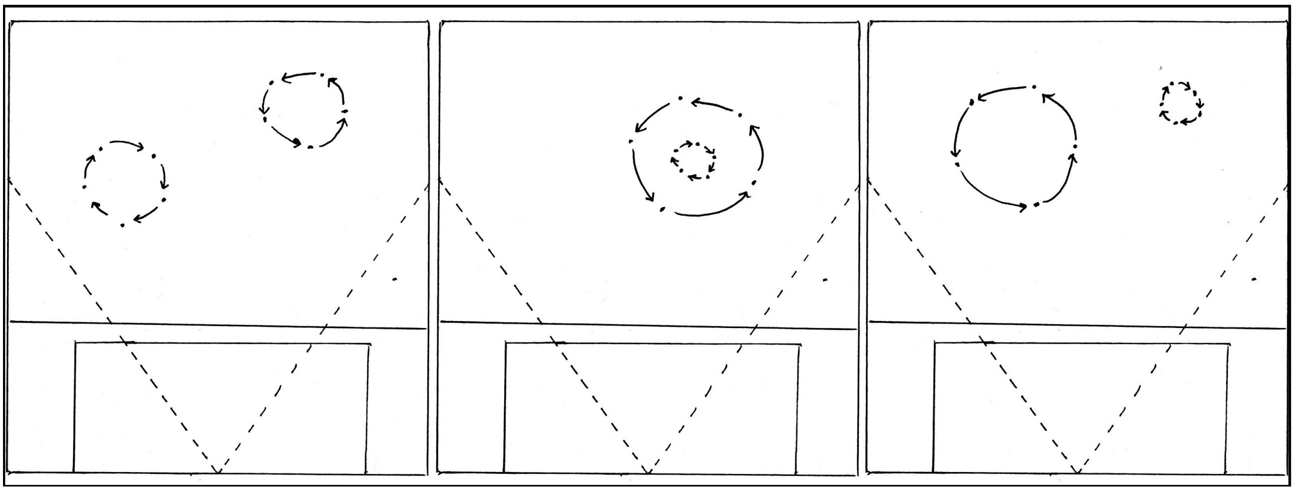

Begin to choose a moment to make a switch from one circle to the other. Let the circles stay in motion by gradually changing in size, location and speed. Allow a third (and possibly fourth) small group circle to be formed. Choose a moment to begin your individual circle, until there are ten individual circles in the room. Allow your individual circle to play with a wide range of speeds in particular, as well as becoming small and large, and gliding its centre point far across the floor. Give time for this to continue.

Falling past:

Falling past begins when two people walking towards one another both decide to suspend and fall past one another with their weight on two feet, when they are shoulder-to-shoulder in space. Gradually, the number of people engaging in falling past and the frequency of falls in the space increases. The group begins to work toward all falling at the same moment in time. A fall is always triggered by two people who fall past each other when they are shoulder-to-shoulder in space, but the rest of the group can join in their fall from anywhere in space. When the whole group successfully falls together at the same moment, everyone calls 'stop' and there is an abrupt standing stillness.

Falling:

Falling[4] begins, with two further 'stops'. Gradually, the first set of materials are brought into play in your continually changing composition: 'stop', 'short walk', 'run', 'long walk'. Once these materials have an opportunity to become familiar to the audience, 'fall' and 'wildcard' are also introduced. There is an invitation for the amount of time between falls to be varied. The work is on following your interest and playing collectively with the relationship between predictability and unpredictability in the composition for the audience.

Exit:

When you notice the end of your composition, you exit, leaving your numbered top behind. The last person(s) takes their time to allow their composition to find its ending.

Performed on 22 & 23 May, 2015 at Brunel University London in the context of scientific research investigating the aesthetic and social effects of synchronous movement of groups.

Concept: Guido Orgs and Matthias Sperling

Choreography, score: Matthias Sperling

Performers: Ben Ash, Andrea Buckley, Antonio de la Fe, Kip Johnson, Michael Kitchin, Evangelia Kolyra, Annie Pui Ling Lok, Rosalie Wahlfrid, Sara Wookey, Robert Vesty

© Matthias Sperling 2015

Endnotes

[1] ‘What lights up’ refers to noticing an area that is highlighted in one’s awareness of one’s own body in a given moment, and initiating movement from that specific place. It may be located on the surface of one’s body or internally; the area noticed may be large or small; more than one area at a time may ‘light up’ and be used.

[2] The basic twisting action used at the beginning and end of this task is a repeated side-to-side twist of the upper body and loosely hanging arms, over a still lower body. The hands remain near hip-height as the arms swing freely away from the body a little with the momentum of the twist, and then gently land, wrapping around the torso, at the extremity of the twist. The hips and knees remain in a constant forward direction, and knees are slightly bent, helping the action to remain grounded. The eyes are what leads the twist around from side to side, allowing performers to see one another regardless of their spatial orientation.

[3] During the rehearsal process, sports bibs numbered between one and ten are distributed randomly to the performers, for use as an element of costuming. Whatever number bib a performer has on that occasion becomes their individual number to be spoken during this count down, and remains so, even when the bibs are redistributed differently amongst the performers for each subsequent performance (to counteract the possibility that audience liking of individual performers is influenced by what number they are wearing).

[4] The ‘Falling’ task is based on the whole group standing still, distributed anywhere in the space and facing any direction, then slowly tipping the whole body forward in one piece. Each time, the aim is for the whole group to share the exact moment of losing control of their weight and experiencing a split-second of free-fall. At each moment of free-fall, each performer makes a choice about which of several movement options they will follow the fall with, and they shout the name of this choice out loud. Performers have the option to occasionally not make a choice themselves, but rather hear what another’s choice is and join in that activity with them.
The options of movement materials are:
‘stop’ (gather your weight and return to standing as quickly and efficiently as possible);
‘short walk’ (an easy walking pace, with a duration based on a roughly shared sense of what ‘short’ means amongst the group, developed during rehearsals);
‘run’ (a quick jogging pace, having a similar duration to short walk);
‘long walk’ (having the same pace as the short walk, but double the duration, or occasionally longer);
‘fall’ (allow your fall to continue to the floor, stay there until everyone else’s movement choice has reached its conclusion, then stand up to reset for the next fall, while the group waits for you);
‘wildcard’ (any other movement choice that you have the impulse to introduce as a contrast within the group composition, that is a discrete movement idea in terms of image and rhythm - an option to be used sparingly).
The options are introduced in successive stages, as detailed by the score.
The task is to collectively use these individual operations to generate an emergent group composition that continually introduces new variations from the audience’s perspective.
